# Major Contribution of Caspase-9 to Honokiol-Induced Apoptotic Insults to Human Drug-Resistant Glioblastoma Cells

**DOI:** 10.3390/molecules25061450

**Published:** 2020-03-23

**Authors:** Gong-Jhe Wu, Sun-Ta Yang, Ruei-Ming Chen

**Affiliations:** 1 Department of Anesthesiology, Shin Kong Wu Ho-Su Memorial Hospital, Taipei 111, Taiwan; m000730@ms.skh.org.tw; 2 Graduate Institute of Medical Sciences, College of Medicine, Taipei Medical University, Taipei 110, Taiwan; 08512@s.tmu.edu.tw; 3 Department of Neurosurgery, School of Medicine, College of Medicine, Taipei Medical University, New Taipei City 235, Taiwan; 4 TMU Research Center of Cancer Translational Medicine, Taipei 110, Taiwan; 5 Anesthesiology and Health Policy Research Center, Taipei Medical University, Taipei 110, Taiwan

**Keywords:** drug-resistant glioblastomas, honokiol, caspase-9, apoptotic insults

## Abstract

Temozolomide (TMZ)-induced chemoresistance to human glioblastomas is a critical challenge now. Our previous studies showed that honokiol, a major bioactive constituent of *Magnolia officinalis* (Houpo), can kill human glioblastoma cells and suppresses glioblastoma growth. This study was further aimed to evaluate the effects of honokiol on human drug-resistant glioblastoma cells and the possible mechanisms. The results by data mining in the cancer genome atlas (TCGA) database and immunohistochemistry displayed that expression of caspase-9 mRNA and protein in human glioblastomas was induced. Human TMZ-resistant U87-MG-R9 glioblastoma cells were selected and prepared from human drug-sensitive U87-MG cells. Compared to human drug-sensitive U87-MG cells, TMZ did not affect viability of U87-MG-R9 glioblastoma cells. Interestingly, treatment with honokiol suppressed proliferation and survival of human drug-resistant glioblastoma cells in concentration- and time-dependent manners. Compared to caspase-8 activation, honokiol chiefly increased activity of caspase-9 in U87-MG-R9 cells. Successively, levels of cleaved caspase-3 and activities of caspase-3 and caspase-6 in human TMZ-tolerant glioblastoma cells were augmented following honokiol administration. In parallel, honokiol triggered DNA fragmentation of U87-MG-R9 cells. Accordingly, treatment of human TMZ-resistant glioblastoma cells with honokiol induced cell apoptosis but did not affect cell necrosis. Fascinatingly, suppressing caspase-9 activity using its specific inhibitors repressed honokiol-induced caspase-6 activation, DNA fragmentation, and cell apoptosis. Taken together, this study has shown the major roles of caspase-9 in transducing honokiol-induced mitochondria-dependent apoptosis in human drug-resistant glioblastoma cells. Thus, honokiol may be clinically applied as a drug candidate for treatment of glioblastoma patients with chemoresistance.

## 1. Introduction

Glioblastomas are the most common and aggressive brain tumors [1]. The patients with malignant glioblastomas usually have poor prognosis [2]. The median survival rate of glioblastoma patients is about 12 months. Temozolomide (TMZ) is the first-line chemotherapeutic drug for therapy of malignant glioblastomas [3]. Nevertheless, TMZ may induce drug tolerance to high-grade glioblastomas, especially in recurrent patients [4]. Thus, the chemoresistance is a serious issue for therapy of human glioblastomas. To build up a novel and effective strategy for treatment of human glioblastomas, it is very critical to discover new chemotherapeutic drugs that can overcome TMZ-induced drug tolerance.

Honokiol (2-(4-hydroxy-3-prop-2-enyl-phenyl)-4-prop-2-enyl-phenol), a small-molecule bisphenol polyphenol, is a major bioactive constituent of the traditional Chinese medicine *Magnolia officinalis* (Houpo) [5]. Amorati et al. demonstrated that the hydroxyl group of the second phenol possesses better chemical reactivity with peroxyl radicals [6]. Honokiol can effectively treat a variety of diseases, including anxiety and nervous disturbances, thrombotic stroke, typhoid fever, and dermatologic disorders [5]. Drug resistance to therapy in cancer is multifaceted and challenged until now. Interestingly, Tian et al. demonstrated that honokiol could synergize chemotherapeutic drugs in multidrug resistant breast cancer cells via apoptotic and programmed necrotic death [7]. A previous study used pharmacogenomics and molecular docking approaches to supplementary show epidermal growth factor receptor (EGFR)-transfected tumor cells were collaterally sensitive to honokiol compared with wild type cells [8]. Recently, honokiol is reported to be a promising natural compound in overcoming acquired resistance to cetuximab, a monoclonal antibody against EGFR used for treatment of head and neck squamous cell carcinoma and metastatic colorectal cancer [9]. As a result, targeting drug resistance by using honokiol alone or combined with other chemotherapy agents can provide de novo therapeutic strategies.

A previous study reported low toxicity of honokiol to normal human astrocytes and murine cerebrovascular endothelial cells [10]. The blood-brain barrier (BBB) is the major limitation for therapy of brain diseases [11]. Notably, honokiol was shown to pass through the BBB in vitro and in vivo [10]. Our laboratory reported the benefits of honokiol to induce apoptosis of neuroblastoma cells and glioblastoma cells via an intrinsic mitochondria-dependent pathway [10,12]. Moreover, the molecular mechanisms were confirmed through a p53/phosphoinositide 3-kinases (PI3K)/mammalian target of rapamycin (mTOR) mechanism and an endoplasmic reticular stress/extracellular signal-regulated kinases (ERK)1/2 pathway in neuroblastoma cells and glioblastoma cells, respectively [13,14]. In addition, autophagy induced by cancer therapy frequently contributes to cancer cell survival [15]. The effects of honokiol on autophagy of neuroblastoma cells and glioblastoma cells were further identified [12,13,14,15]. Furthermore, cancer stemness is the other critical cause for drug resistance [16]. Previous studies presented the potential of honokiol on suppressing sphere formation and xenograft growth of oral cancer stem cells [17,18]. Thus, honokiol has the potential for treatment of drug-resistant glioblastomas.

Antiapoptosis of cancer cells against chemotherapy is the other important reason for chemoresistance [19]. Extrinsic and intrinsic pathways are involved in cell apoptosis. In an extrinsic pathway, caspase-8 is activated following binding of extracellular cytotoxic Fas ligand to its death receptor [20]. In contrast, activation of capase-9 by release of mitochondrial cytochrome c to the cytoplasm can trigger apoptosis via an intrinsic mechanism [20,21]. Recently, we have shown that honokiol could synergistically improve TMZ-induced killing to human malignant glioblastoma cells through a mitochondrion-dependent apoptotic mechanism [22,23]. Hence, caspase-8 and caspase-9 are two typical molecules specifically triggering cell apoptosis through an extrinsic death ligand-dependent mechanism and an intrinsic mitochondria-dependent pathway, respectively [20,24]. Based on previous studies, honokiol is able to kill glioblastoma cells by inducing autophagic and apoptotic insults. Elucidating the apoptotic mechanism is crucial for clinical application of honokiol for treatment of drug-resistant glioblastomas. Therefore, this study was aimed to further evaluate the effects of honokiol on the drug-tolerant glioblastoma cells and the possible mechanisms, especially in the caspases-8/-9-involed apoptotic pathways.

## 2. Results

### 2.1. Higher Expression of Caspase-9 in Human Glioblastomas

**Figure 1:**
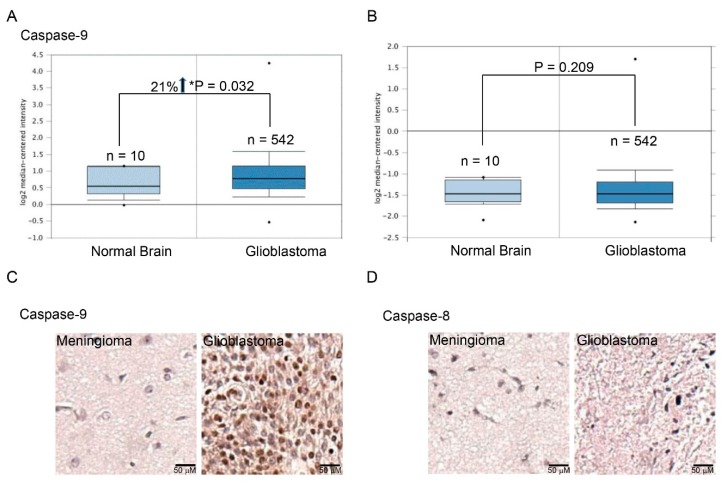
Differential expressions of caspase-9 and -8 mRNAs and proteins in human control brain tissues and glioblastomas. Expressions of caspase-9 (**A**) and caspase-8 mRNAs (**B**) were mined in the cancer genome atlas (TCGA) database. Immunohistological analyses of caspase-9 (**C**) and caspase-8 (**D**) in human meningioma and glioblastoma tissues were further carried out. There were six meningioma and glioblastoma patients checked. The representative images are shown. The symbol * indicates that the value significantly differs from the normal brain group, *p* < 0.05.

Differential expression of caspase-9 and -8 mRNAs in human normal brain tissues and glioblastomas were evaluated using the TCGA cohort (Figure 1). Compared to normal brain tissues, expression of caspase-9 mRNA in human glioblastomas was upregulated by 21% (*p* < 0.05) (Figure 1A). However, expression of caspase-8 mRNA in human normal brain tissues and glioblastomas was not different (*p* = 0.916) (Figure 1B). Immunohistochemical analyses showed augmentation of caspase-9 in human glioblastomas compared to human meningioma tissues (Figure 1C). Levels of caspase-8 in human controls and glioblastomas were not changed (Figure 1D).

### 2.2. Preparation of Human Drug-Resistant Glioblastoma Cells

**Figure 2:**
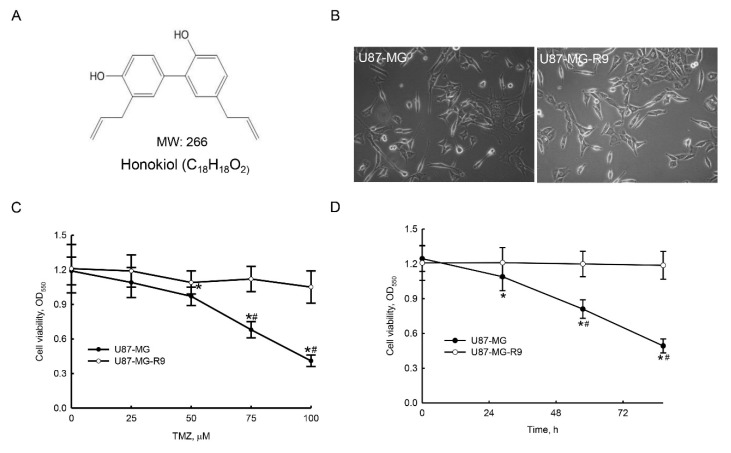
Selection of human drug-resistant glioblastoma cells. Chemical structure and molecular weight (MW) of honokiol is shown (**A**). Human temozolomide (TMZ)-resistant U87-MG-R9 glioblastoma cells were prepared by selection from human U87-MG cells. Morphologies of U87-MG and U87-MG-R9 cells are shown (**B**). Exposure of U87-MG and U87-MG-R9 cells to 25, 50, 75, and 100 μM TMZ for 72 h (**C**) or 100 μM TMZ for 24, 48, and 72 h (**D**). Cell viability was assayed using a colorimetric assay. Each value represents the mean ± SD for at least three independent determinations. The symbols * and # indicates that the value significantly (*p* < 0.05) differs from the control and drug-resistant cells, respectively.

Chemical structure and molecular weight of honokiol is shown in Figure 2A. Human TMZ-resistant U87-MG-R9 glioblastoma cells were prepared by selecting from human malignant U87-MG cells. Morphologies of human U87-MG and U87-MG-R9 glioblastoma cells are shown in Figure 2B. Exposure of human TMZ-sensitive U87-MG cells to 50, 75, 100 μM TMZ for 72 h caused 18%, 43%, and 66% decreases in cell viability, respectively (Figure 2C). However, viability of human TMZ-resistant U87-MG-R9 cells was not altered by TMZ. In addition, exposure of human drug-sensitive glioblastoma cells to 100 μM TMZ for 24, 48, and 72 h led to 13%, 35%, and 60% declines in cell survival (Figure 2D). Treatment of human U87-MG-R9 glioblastoma cells with TMZ did not influence cell survival.

### 2.3. Honokiol Lessened Proliferation and Viability of Human Drug-Resistant Glioblastoma Cells

Compared to the control group, treatment of human TMZ-resistant U87-MG-R9 glioblastoma cells with 20 and 40 µM honokiol for 72 h decreased cell proliferation by 25% and 46%, respectively (Figure S1A). After exposure to 40 µM honokiol for 48 and 72 h later, proliferation of human drug-resistant glioblastoma cells was diminished by 23% and 44% (Figure S1B). Exposure of human TMZ-resistant U87-MG-R9 glioblastoma cells to 20 and 40 µM honokiol for 72 h caused 19% and 36% decreases of cell viability (Figure S1C). In contrast, 48 and 72 h later, treatment with honokiol led to 26% and 38% reductions in viability of human U87-MG-R9 glioblastoma cells (Figure S1D).

### 2.4. Honokiol Mainly Triggered Caspase-9 Activation in Human Drug-Resistant Glioblastoma Cells

**Figure 3:**
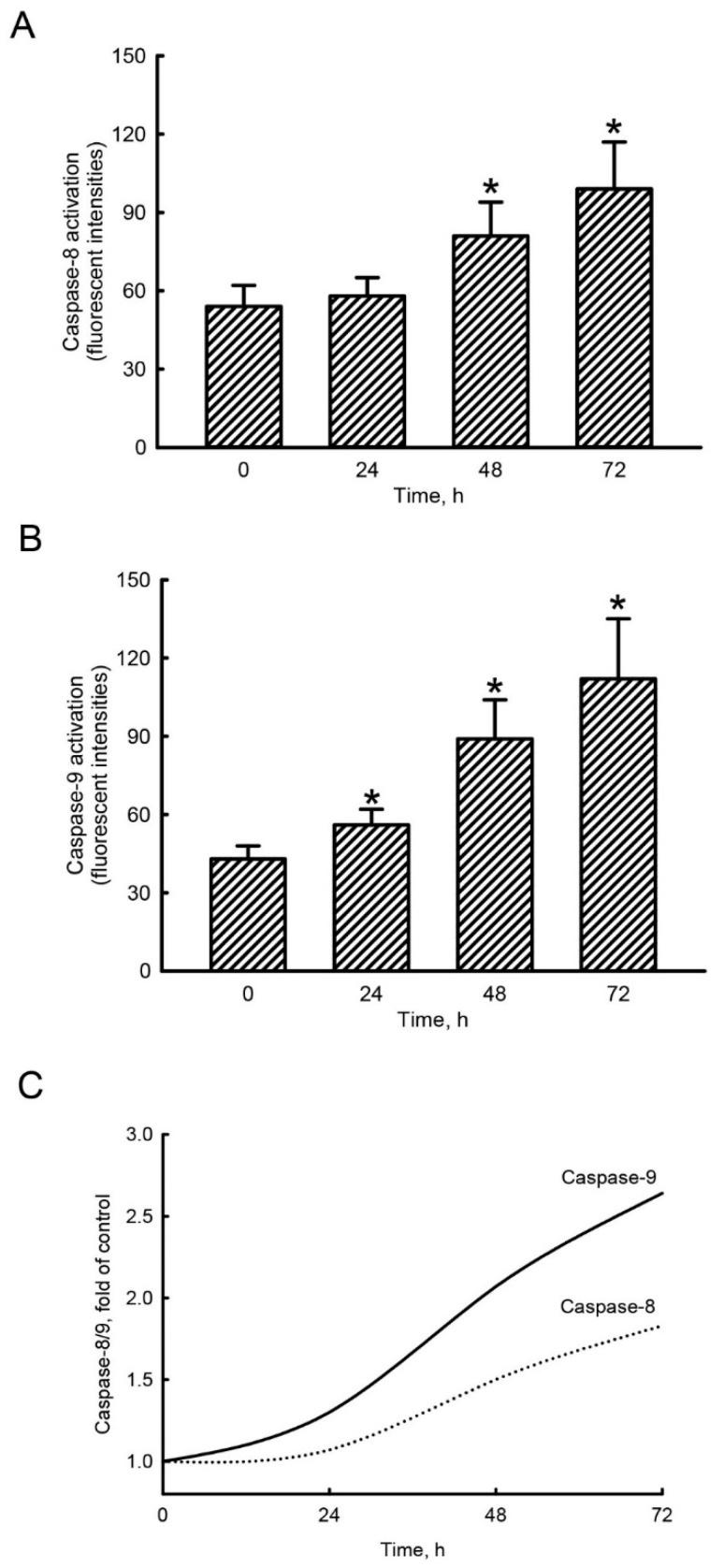
Honokiol mainly induced activation of caspase-9 in human drug-resistant glioblastoma cells. Human TMZ-resistant U87-MG-R9 glioblastoma cells were exposed to 40 μM honokiol for 24, 48, and 72 h. Activities of caspase-8 (**A**) and -9 (**B**) were measured using fluorogenic substrate methods. Effects of honokiol on induction of caspase-8 and caspase-9 were compared cells (**C**). Each value represents the mean ± SD for at least three independent determinations. The symbol * indicates that the value significantly differs from the respective control, *p* < 0.05.

Exposure of human TMZ-resistant U87-MG-R9 glioblastoma cells to 40 µM honokiol for 24 h did not change activity of caspase-8 (Figure 3A). At 48 and 72 h following treatment with honokiol, caspase-8 activity was augmented by 50% and 83%, respectively. In contrast, exposure of human U87-MG-R9 glioblastoma cells to 40 µM honokiol for 24, 48, and 72 h led to significant 30%, 107%, and 160% increases in activity of caspase-9, respectively (Figure 3B). The honokiol-induced activation of caspase-8 and caspase-9 was compared (Figure 3C). In comparison with caspase-8 activation, honokiol induced more 21%, 38%, and 44% stimulation of caspase-9 activity in human malignant drug-resistant glioblastoma cells. In the control groups, activities of caspase-8 and caspase-9 in human drug-resistant glioblastoma cells were not changed after treatment with DMSO for 24, 48, and 72 h.

### 2.5. Honokiol Sequentially Stimulated Cascade Activation of Caspase-3 and Caspase-6 in Human Drug-Resistant Glioblastoma Cells

**Figure 4:**
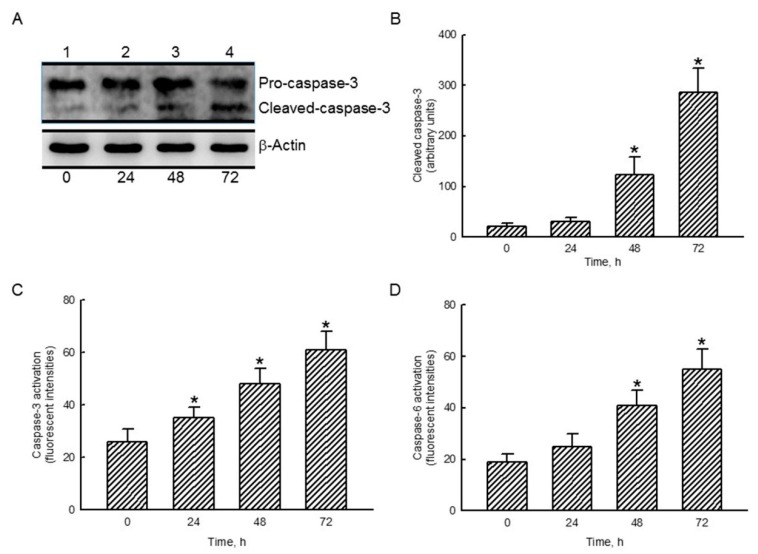
Honokiol induced sequential activation of caspase-3 and caspase-6 in human drug-resistant glioblastoma cells. Human TMZ-resistant U87-MG-R9 glioblastoma cells were exposed to 40 μM honokiol for 24, 48, and 72 h. Levels of pro- and cleaved caspase-3 were immunodetected (**A**, top panel). β-Actin was analyzed as an internal control (bottom panel). These protein bands were quantified and statistically analyzed (**B**). Activities of caspase-3 (**C**) and caspase-6 (**D**) were assayed using fluorogenic substrate methods. Each value represents the mean ± SD for at least three independent determinations. The symbol * indicates that the value significantly differs from the respective control, *p* < 0.05.

In untreated human TMZ-resistant U87-MG-R9 glioblastoma cells, low levels of cleaved caspase-3 were detected (Figure 4A, top panel, lane 1). Compared to the control group, exposure to 40 µM honokiol for 24 h slightly elevated amounts of cleaved caspase-3 (lane 2). In contrast, treatment of U87-MG-R9 cells with honokiol for 48 and 72 h apparently increased levels of cleaved caspase-3 (lanes 3 and 4). Amounts of β-actin were analyzed as the internal controls (bottom panel). These protein bands were quantified and statistically analyzed (Figure 4B). Treatment of U87-MG-R9 cells with 40 µM honokiol for 48 and 72 h caused 5.9- and 13.6-fold enhancements in levels of cleaved caspase-3 (Figure 4B). Exposure of human U87-MG-R9 cells to 40 µM honokiol for 24, 48, and 72 h caused 35%, 85%, and 135% augmentations in caspase-3 activity, respectively. (Figure 4C). Twenty-four hours later, honokiol at 40 µM did not influence caspase-6 activity compared to untreated cells. However, treatment of U87-MG-R9 cells with honokiol for 48 and 72 h led to 2.2- and 2.9-fold increases in caspase-6 activities, respectively (Figure 4D).

### 2.6. Honokiol Selectively Induced DNA Fragmentation and Cell Apoptosis in Human Drug-Resistant Glioblastoma Cells

**Figure 5:**
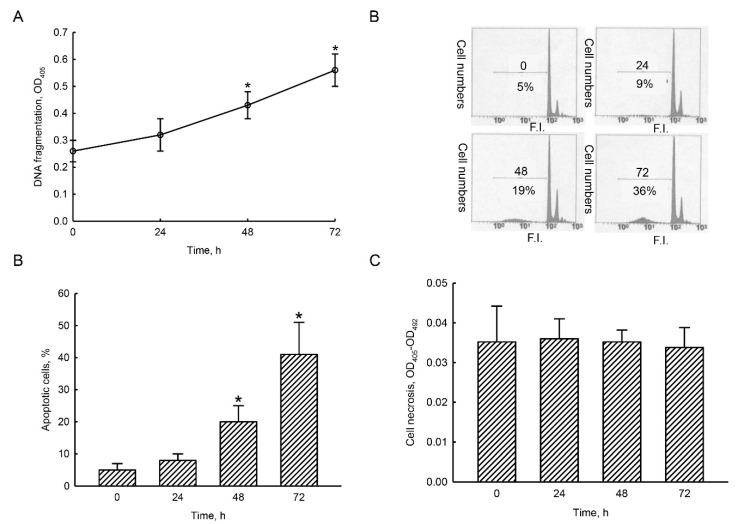
Honokiol consequently induced DNA fragmentation and cell apoptosis but not cell necrosis in human drug-resistant glioblastoma cells. Human TMZ-resistant U87-MG-R9 glioblastoma cells were exposed to 40 μM honokiol for 24, 48, and 72 h. DNA fragmentation was quantified with a cellular DNA fragmentation ELISA kit (**A**). Analysis of cell cycle was conducted using flow cytometry (**B**). Percentages of the cells at sub-G1 phase were quantified and statistically analyzed (**C**). Cell necrosis was assayed by using a photometric immunoassay (**D**). Each value represents the mean ± SD for at least three independent determinations. The symbol * indicates that the value significantly differs from the respective control, *p* < 0.05. F.I., fluorescent intensities.

Exposure of human TMZ-resistant glioblastoma cells to 40 µM honokiol for 24 h did not affect DNA fragmentation (Figure 5A). In contrast, 48 and 72 h later, honokiol induced DNA fragmentation by 65% and 115%, respectively. The results by flow cytometry revealed that treatment of U87-MG-R9 cells with 40 µM honokiol for 24 h did not change the proportion of the cells at the sub-G1 phase compared to the untreated cells (Figure 5B). In contrast, the percentages of human TMZ-resistant glioblastoma cells at sub-G1 phase were obviously elevated following honokiol administration. These data were quantified and statistically analyzed (Figure 5C). Honokiol did not trigger apoptosis of human U87-MG-R9 glioblastoma cells following exposure for 24 h. However, treatment with 40 µM honokiol for 48 and 72 h caused 20% and 41% expansions in apoptosis of human TMZ-resistant U87-MG-R9 glioblastoma cells, respectively (Figure 5C). Compared to the control group, exposure of human TMZ-resistant U87-MG-R9 glioblastoma cells to 40 µM honokiol for 24 h did not trigger cell necrosis (Figure 5D). Forty-eight and 72 h later, proportions of human U87-MG-R9 cells undergoing necrosis were not changed.

### 2.7. Caspase-9 Contributes to Honokiol-Induced Caspase-6 Activation, DNA Fragmentation, and Cell Apoptosis in Human Drug-Resistant Glioblastoma Cells

**Figure 6:**
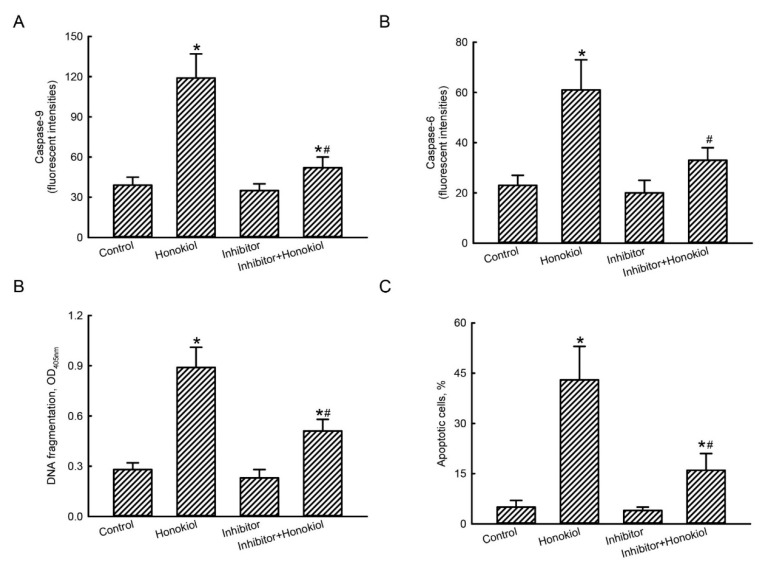
Involvement of caspase-9 in honokiol-induced apoptotic insults to human drug-resistant glioblastoma cells. Human TMZ-resistant U87-MG-R9 glioblastoma cells were pretreated with inhibitors of caspase-9 for 1 h and then exposed to 40 μM honokiol for 72 h. Activities of caspase-9 (**A**) and caspase-6 (**B**) were assayed using fluorogenic substrate methods. DNA fragmentation was quantified with an ELISA kit (**C**). Apoptotic cells were measured using flow cytometry (**D**). Each value represents the mean ± SD for at least three independent determinations. The symbol * and # indicate that the value significantly (*p* < 0.05) differs from control and honokiol-treated groups, respectively.

Exposure of human TMZ-resistant glioblastoma cells to 40 µM honokiol for 72 h led to a 3.1-fold elevation in the activity of caspase-9 (Figure 6A). Pretreated with caspase-9 inhibitors suppressed honokiol-induced activation of caspase-9 by 84%. Activities of caspase-6 were raised by 2.7-fold in U87-MG-R9 cells following administration of honokiol (Figure 6B). Repressing caspase-9 activity concurrently decreased honokiol-triggered caspase-6 activation. Exposure of U87-MG-R9 cells to honokiol induced DNA fragmentation and cell apoptosis by 3.2- and 8.6-fold, respectively (Figure 6C,D). Reduction of caspase-9 activity consequently repressed honokiol-induced DNA fragmentation and cell apoptosis in human drug-resistant U87-MG-R9 glioblastoma cells (Figure 6C,D).

## 3. Discussion

In this study, we have shown that honokiol could kill the drug-resistant glioblastoma cells. Glioblastomas are major solid brain tumors [1]. Until now, administration of TMZ may lead to drug resistance, especially for high-grade glioblastomas and recurrent patients [4]. In the present investigation, we successfully prepared human drug-tolerant U87-MG-R9 glioblastoma cells from human U87-MG cells as our experimental model. In drug discovery for therapy of brain tumors, the BBB is a significant limitation and challenge [10]. Fascinatingly, our previous study has demonstrated that honokiol could pass through the BBB and possess low toxicity to normal human HA-h astrocytes and mouse cerebrovascular endothelial cells [10,22]. Furthermore, we reported the benefits of propofol on killing human U87-MG and U373MG and murine GL-261 glioblastoma cells, as well as neuroblastoma cells. Recently, we demonstrated the synergistic effects of honokiol on TMZ-induced insults to glioblastoma cells [22,23]. In this study, we further verify the welfares of honokiol to efficiently kill human TMZ-resistant glioblastoma cells. Human malignant glioblastomas are very aggressive and recurrent [2]. Most of glioblastoma patients are drug-resistant and recurrent [3,4]. Cancer stemness and autophagy are two key causes for drug resistance. A previous study reported the suppressive effects of honokiol on sphere formation and xenograft growth of oral cancer stem cells [17]. In addition, honokiol can induce autophagic insults to neuroblastoma cells and glioblastoma cells [13,14]. As a result, our study attractively suggested that honokiol has potential welfares for therapy of drug-resistant glioblastoma patients.

Rapid proliferation is an important characteristic of malignant glioblastoma cells [1,25]. Furthermore, rapid tumor growth because of speedy cell proliferation is one of major features and reasons illuminating the casual recurrence and poor prognoses of human malignant glioblastomas [26]. Herein, we demonstrated that honokiol could descend proliferation of human drug-resistant glioblastoma cells. The honokiol-induced suppression in proliferation of human TMZ-resistant glioblastoma cells discloses the other potential benefits for therapy of malignant glioblastomas. In tumor microenvironments, low oxygen conditions are broadly present [27]. Our previous study showed that under extended hypoxic stress, proliferation of human U87-MG cells was significantly repressed [28]. A previous study has also reported that hypoxia is able to activate a self-protective mechanism against glioblastoma proliferation [29]. Consequently, honokiol can prevent tumor cell proliferation in a hypoxic microenvironment and then suppress tumor growth.

Honokiol can activate caspase-3 and caspase-6 in human drug-resistant glioblastoma cells. Caspase-3, a protease in the process of intrinsic and extrinsic apoptosis, is activated by caspase-9 [12]. Sequentially, treatment with honokiol enhanced caspase-6 activity, a downstream target of caspase-3, in human drug-resistant glioblastoma cells. Activations of caspase-9 and capse-3 are essential for proteolytic maturation of caspase-6 [30]. After activation, caspase-3 and caspase-6 can cleave cellular crucial proteins such as lamin and nuclear mitotic apparatus proteins to affect cell functions [31]. Our previous study has shown that honokiol could stimulate cascade activations of caspase-9 and -3, G1 phase arrest, and cell apoptosis in human TMZ-sensitive glioblastoma cells [12]. In the present study, honokiol could increase levels of cleaved caspase-3 and activity of this proteinase. Our previous and present studies displayed the helpful effects of honokiol on killing drug-sensitive and -resistant glioblastoma cells through cascade activation of the caspase proteases.

Honokiol can kill human drug-resistant glioblastoma cells via an apoptotic pathway due to a main activation of caspase-9. Our present study revealed that honokiol could induce caspase activation and DNA fragmentation in human TMZ-resistant glioblastoma cells. In addition, treatment with honokiol led to cell shrinkage and cell cycle arrest at the sub-G1 phase. Characteristically, a shrunken morphology, caspase activation, DNA fragmentation, and cell cycle arrest at the sub-G1 phase are significant features of cells undergoing apoptosis [32]. As a result, honokiol can meaningfully kill human drug-resistant glioblastoma cells via an apoptotic pathway. Antiapoptosis of cancer cells against chemotherapy is another important cause resulting in drug resistance [19]. Jeong et al. reported the anticancer effects of honokiol on human glioblastoma cells via induction of cell apoptosis [33]. Moreover, a previous study also demonstrated the effects of honokiol on triggering apoptosis of glioblastoma cells via p53/p21-mediated cell cycle arrest at the G1 phase [13]. Zhang et al. stated that honokiol induced apoptosis of U87-MG cells via activation of p38MAPK [34]. In addition, our previous study further showed that honokiol can repress growth of human glioblastomas by inducing cell apoptosis due to activating the p53/cyclin D1/CDK6/CDK4/E2F1-dependent mechanism [12]. Caspase-8 and -9 are two typical molecules explaining the extrinsic and intrinsic mechanisms of cell apoptosis [20,24]. In this study, we showed that exposure to honokiol triggered more activation of caspase-9 than caspase-8 in human drug-resistant glioblastoma cells. In addition, our data mining in TCGA cohort further verified higher expression of caspase-9 mRNA in human glioblastomas. In addition, levels of caspase-9 protein in human glioblastomas were enlarged compared to human meningioma tissues. Interestingly, knocking-down caspase-9 concurrently lowered honokiol-induced caspase-6 activation, DNA damage, and cell apoptosis. As a result, caspase-9 plays a major role in honokiol-induced apoptotic insults to human drug-resistant glioblastoma cells.

Drug resistance to therapy in cancer is multifaceted and challenged until now. Antiapoptosis of cancer cells against chemotherapy is an important reason for chemoresistance. Honokiol is a multifunctional antiangiogenic and antitumor agent [5]. Compared with wild type cells, Saeed et al. reported that that the EGFR-transfected tumor cells were collaterally sensitive to honokiol [8]. In multidrug resistant breast cancer cells, honokiol can synergize chemotherapeutic drugs to induce apoptotic and programmed necrotic cell death [7]. Lately, honokiol is reported to be a promising natural compound in overcoming acquired resistance to cetuximab, a monoclonal antibody against EGFR used for treatment of head and neck squamous cell carcinoma and metastatic colorectal cancer [9]. Our previous study has demonstrated synergistic effects of honokiol with TMZ to kill human glioblastoma cells [23]. In this study, we further showed a major role of caspase-9 in mediating honokiol-induced apoptotic insults to human drug-resistant glioblastoma cells. Therefore, honokiol can be a drug candidate for treatment of glioblastoma patients with chemoresistance.

## 4. Materials and Methods

### 4.1. Data Downloading and Preprocessing

Expressions of caspase-8 and caspase-9 mRNAs in human normal brain tissues and glioblastomas were analyzed by searching the cancer genome atlas (TCGA) database (https://www.cancer.gov/about-nci/organization/ccg/research/structural-genomics/tcga, data download time December 2019). mRNA expression data were selected for downloading in the FPKM format.

### 4.2. Immunohistochemical Analyses of Caspase-8 and Caspase-9

Our study was approved by the joint-institutional review board of Taipei Medical University. Specimens of human meningioma and glioblastoma tissues were identified and acquired from the Department of Pathology, Shuang Ho Hospital, Taipei Medical University, Taipei, Taiwan. These brain specimens were incubated with 0.2% Triton X-100. Mouse monoclonal antibodies against human caspase-8 and caspase-9 (Santa Cruz Biotechnology, Santa Cruz, CA, USA) were used for immunodetections of caspase-8 and caspase-9, as described previously [35].

### 4.3. Cell Culture

Human U87-MG glioblastoma cells, purchased from American Type Culture Collection (Manassas, VA, USA), were seeded in Dulbecco’s modified Eagle’s medium (DMEM; Gibco-BRL Life Technologies, Grand Island, NY, USA) supplemented with 10% fetal bovine serum (FBS), L-glutamine (2 mM), penicillin (100 IU/mL), streptomycin (100 mg/mL), sodium pyruvate (1 mM), and nonessential amino acids (1 mM) at 37 °C in a humidified atmosphere of 5% CO2. The drug-sensitive glioblastoma cells were grown to confluence before drug treatment, as described previously [36].

### 4.4. Preparation of Human Drug-Resistant Glioblastoma Cells and Drug Treatment

Human TMZ-resistant glioblastoma cells were prepared and selected, as described previously [37]. TMZ was purchased from Enzo Life Sciences (Farmingdale, NY, USA) and was dissolved in dimethyl sulfoxide (DMSO). Human U87-MG cells (10^5^) were seeded in 12-well tissue culture plates and maintained in a culture medium supplemented with TMZ at 50 μM. Two days later, U87-MG cells were subsequently dissociated, seeded at 1/5~1-fold dilutions in 96-well tissue culture plates, and then maintained in a culture medium with 100 μM TMZ. When a surviving colony produced, cells were dissociated with trypsin and allowed to grow in the same well. Among these drug-resistant colonies, colony 9 (U87-MG-R9) was more malignant and had a more-rapid growth rate. Honokiol, purchased from Sigma (St. Louis, MO, USA), was freshly dissolved in DMSO. The purity of honokiol used in this study was more than 98%. Human U87-MG and U87-MG-R9 cells were exposed to honokiol, TMZ at 100 μM, and a combination of honokiol and TMZ for various time intervals. Control cells received DMSO (less than 0.1%).

### 4.5. Analysis of Cell Proliferation

Cell proliferation was analyzed by counting cell number, as described previously [38]. Briefly, human U87-MG-R9 cells (2 × 10^4^ cells) were cultured in 24-well tissue culture plates. After treatment with honokiol, the glioblastoma cells were trypsinized with 0.1% trypsin-EDTA (Gibco-BRL). Following centrifugation and washing, the cells were suspended in a phosphate-based saline buffer (PBS, 0.14 M NaCl, 2.6 mM KCl, 8 mM Na2HPO4, and 1.5 mM KH2PO4) and stained with trypan blue dye (Sigma). Cell numbers were counted using a reverse-phase microscope (Nikon, Tokyo, Japan).

### 4.6. Assay of Cell Viability

Cell viability was assayed using a colorimetric method as described previously [39]. Human U87-MG-R9 cells were seeded in 96-well tissue culture plates at a density of 1 ×10^4^ cells per well overnight. After treatment with honokiol, the cells were cultured in new medium containing 3-(4,5-dimethylthiazol-2-yl)-2,5-diphenyltetrazoliumbromide (0.5 mg/mL) for a further 3 h. The blue formazan products in cells were dissolved in DMSO, and the optical densities were spectrophotometrically measured at a wavelength of 550 nm.

### 4.7. Assay of Caspase Activities

Activities of caspase-3 and caspase-6 were assayed following a previously described method [40,41]. After drug treatment, human TMZ-resistant U87-MG-R9 glioblastoma cells were lysed. Cell extracts (25 mg of total protein) were incubated with 50 mM of a specific fluorogenic peptide substrate in 200 mL of a cell-free system buffer. The peptide substrates for the caspase-3, -6, 8, and -9 assays were DEVD, VEID, IETD, and LEHD, respectively. The peptides were conjugated to 7-amino-4-trifluoromethyl coumarin for fluorescence detection. Intensities of the fluorescent products were measured with a spectrometer.

### 4.8. Immunoblotting Analyses

Pro- and cleaved caspase-3 were analyzed using an immunoblotting method as described previously [42]. After drug treatment, human U87-MG-R9 cells were washed with PBS and lysed with an ice-cold lysis buffer (25 mM HEPES, 1.5% Triton X-100, 0.1% sodium dodecylsulfate (SDS), 0.5 M NaCl, 5 mM EDTA, and 0.1 mM sodium deoxycholate containing a protease inhibitor cocktail, including leupeptin (10 mg/mL), aprotinin (0.27 U/mL), and phenylmethylsulfonyl fluoride (PMSF, 100 mm). Protein concentrations were quantified using a bicinchonic acid protein assay kit (Thermo, San Jose, CA, USA). Cellular proteins were separated using SDS-polyacrylamide gel electrophoresis (SDS-PAGE) and then transferred to nitrocellulose membranes. Following blocking with a 5% skim milk, the membranes were incubated with a caspase-3 antibody (Cell Signaling Technology, Beverly, MA, USA). Membranes were probed with the appropriate horseradish peroxidase-conjugated secondary antibodies. Levels of β-actin protein was immunodetected using a mouse monoclonal antibody (Sigma) as the internal standard. Immunoreactive proteins were detected using an enhanced chemiluminescence reagent (PerkinElmer, Waltham MA, USA) and then imaged using a digital analyzer (Syngene, Cambridge, UK) and a densitometry software (Syngene).

### 4.9. Quantification of DNA Fragmentation

DNA fragmentation in human U87-MG glioblastoma cells was quantified using a cellular DNA fragmentation enzyme-linked immunosorbent assay (ELISA) kit (Boehringer Mannheim, Indianapolis, IN, USA), as described previously [43]. Briefly, 2 × 10^5^ human U87-MG-R9 glioblastoma cells were subcultured in 24-well tissue culture plates and labeled with 5-bromo-2′-deoxyuridine (BrdU) overnight. The drug-resistant glioblastoma cells were harvested and suspended in culture medium. One hundred microliters of the cell suspension were added to each well of 96-well tissue culture plates. Human U87-MG-R9 cells were cocultured with honokiol and TMZ for another 8 h at 37 °C in a humidified atmosphere of 5% CO2. Amounts of BrdU-labeled DNA in the cytoplasm were quantified using an Anthos 2010 microplate photometer (Anthos Labtec Instruments, Lagerhausstrasse, Wals/Salzburg, Austria) at a wavelength of 450 nm.

### 4.10. Analysis of Apoptotic Cells

Apoptosis of human TMZ-resistant U87-MG-R9 glioblastoma cells were quantified by using propidium iodide (PI) to detect DNA injury in nuclei according to a previously described method [12]. After treatment with honokiol, human drug-resistant glioblastoma cells were harvested and then fixed in cold 80% ethanol. Following centrifugation and washing, fixed cells were stained with PI and analyzed using a FACScan flow cytometer (EPICS XL, Beckman Coulter, Fullerton, CA, USA).

### 4.11. Quantification of Necrotic Cells

Necrotic cells were quantified using a photometric immunoassay according to a previously described method [10]. Briefly, human U87-MG-R9 glioblastoma cells (1 × 10^5^ cells) were seeded in 96-well tissue culture plates overnight. After drug administration, cell lysates and culture medium were collected, and necrotic cells were immunodetected using mouse monoclonal antibodies against histone. After an antibody reaction and washing, the colorimetric product was measured at 405 nm against a substrate solution as a blank. DMSO at 1 mM was administered into human TMZ-resistant glioblastoma cells for different time intervals as a positive control and the necrotic analysis was then carried out.

### 4.12. Inhibition Assay of Caspase-9 Activity

For the inhibition assay, human drug-resistant U87-MG-R9 glioblastoma cells were pretreated with Z-LEHD-FMK, an inhibitor of caspase-9, at 50 mM for 1 h, and then exposed to honokiol for 72 h following the method described previously [15]. Intensities of the fluorescent products were measured using a LS 55 spectrometer of PerkinElmer Instruments (Shelton, CT, USA).

### 4.13. Statistical Analyses

The statistical significance of differences between groups was evaluated using a one-way analysis of variance (ANOVA) with Duncan’s multiple-range test. Differences were considered statistically significant at *p*-values of < 0.05. Each value represents the mean ± SD.

## 5. Conclusions

This study has successfully selected human TMZ-resistant U87-MG-R9 glioblastoma cells from human malignant U87-MG cells. We used the drug-tolerant glioblastoma cells as our experimental model to verify the beneficial effects of honokiol on suppressing cell proliferation and decreasing cell viability. As for the mechanisms, we showed that treatment of human TMZ-resistant U87-MG-R9 glioblastoma cells with honokiol did not lead to cell necrosis. Instead, honokiol could stimulate activation of caspase-3 and caspase-6 in human drug-tolerant glioblastoma cells. Successively, honokiol could induce DNA fragmentation in a time-dependent manner. Consequently, proportions of human U87-MG-R9 glioblastoma cells undergoing apoptosis were significantly augmented following treatment with honokiol. Fascinatingly, honokiol induced a major activation of caspase-9. The results by mining TCGA database and immunohistochemistry also showed upregulation of caspase-9 mRNA and protein in human glioblastomas. Therefore, this study shows that honokiol can kill human TMZ-resistant glioblastoma cells via an apoptotic mechanism concurrently due to a main caspase-9-involved intrinsic pathway. Honokiol may have the benefits for therapy of drug-resistant glioblastoma patients. However, there are certain study limitations in this study. More drug-resistant glioblastoma cell lines and patient-derived drug-resistant glioblastoma cells as the experimental model should be tested in order to confirm our findings. In addition to apoptosis, more death mechanisms such as autophagy, ferroptosis, mitoptosis, and necroptosis should be determined in the future. Moreover, animal studies are panned to be performed in order to confirm our in vitro findings in our next studies.

## Supplementary Materials

The following are available online at https://www.mdpi.com/1420-3049/25/6/1450/s1, Figure S1. Effects of honokiol on proliferation and viability of human malignant drug-resistant glioblastoma cells.
