# Supplementary material for: Major Contribution of Caspase-9 to Honokiol-Induced Apoptotic Insults to Human Drug-Resistant Glioblastoma Cells
Source: Molecules. 2020 Mar 23;25(6):1450. doi: 10.3390/molecules25061450 (PMC7145301; doi:10.3390/molecules25061450)
Supplement: Supplementary file 1 [file molecules-25-01450-s001.pdf]

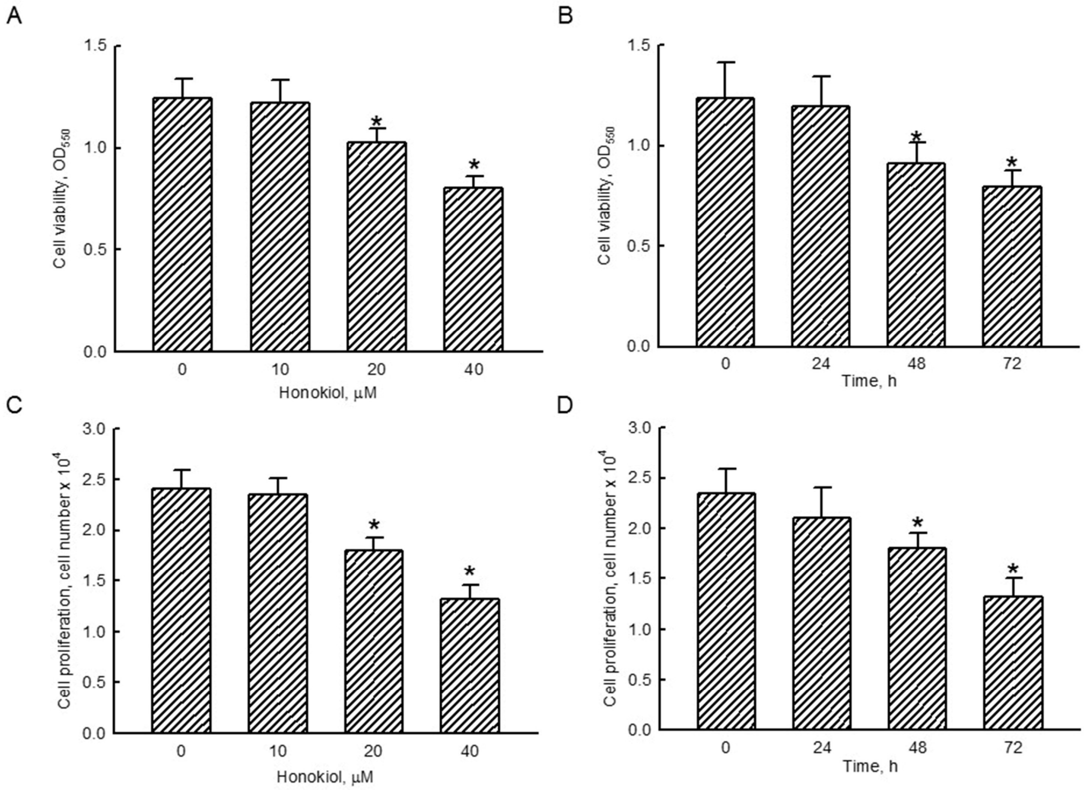

Figure S1. Effects of honokiol on proliferation and viability of human malignant drug-resistant glioblastoma cells. Human temozolomide (TMZ)-resistant U87-MG-R9 glioblastoma cells were exposed to 10, 20, and 40 μM honokiol for 72 h (**A** and **C**) or 40 μM honokiol for 24, 48, and 72 h (**B** and **D**). Cell proliferation was assayed by counting living cells using a trypan blue exclusion method (**A** and **B**). Cell viability was assayed using a colorimetric assay (**C** and **D**). Each value represents the mean ± SEM for $n = 6$. The symbol * indicates that the value significantly differs from the respective control, $p < 0.05$.
